# Immediate and short-term pain relief by acute sciatic nerve press: a randomized controlled trial

**DOI:** 10.1186/1471-2253-7-4

**Published:** 2007-05-16

**Authors:** Jiman He, Bin Wu, Wenlong Zhang, Guangping Ten

**Affiliations:** 1 Biomedicine (TC), Chinese Academy of Sciences, Beijing, 100080 China & Rhode Island Hospital, Brown University, 02903 USA; 2 Renal Department, Anhui Province Hospital, Anhui Medical University, Hefei, 230001 China; 3 Department of Dentistry, Chuzou Zhongxiyi Hospital, Chuzou, 239000 China; 4 Department of Medicine, Tongling People Hospital, Tongling, 244000 China

## Abstract

**Background:**

Despite much research, an immediately available, instantly effective and harmless pain relief technique has not been discovered. This study describes a new manipulation: a "2-minute sciatic nerve press", for rapid short-term relief of pain brought on by various dental and renal diseases.

**Methods:**

This randomized, single-blind, placebo-controlled trial ran in three hospitals in Anhui Province, China, with an enrollment of 66 out of 111 solicited patients aged 16 to 74 years. Patients were recruited sequentially, by specific participating physicians at their clinic visits to three independent hospitals. The diseases in enrolled dental patients included dental caries, periodontal diseases and dental trauma. Renal diseases in recruits included kidney infections, stones and some other conditions. Patients were randomly assigned to receive the "2-minute sciatic nerve press" or the "placebo press". For the "2-minute sciatic nerve press", pressure was applied simultaneously to the sciatic nerves at the back of the thighs, using the fists while patients lay prone. For the "placebo press", pressure was applied simultaneously to a parallel spot on the front of the thighs, using the fists while patients lay supine. Each fist applied a pressure of 11 to 20 kg for 2 minutes, after which, patients arose to rate pain.

**Results:**

The "2-minute sciatic nerve press" produced greater pain relief than the "placebo press". Within the first 10 minutes after sciatic pressure, immediate pain relief ratings averaged 66.4% (p < 0.001) for the dental patients, versus pain relief of 20% for the placebo press, and, 52.2% (p < 0.01) for the renal patients, versus relief of 14% for the placebo press, in median. The method worked excellently for dental caries and periodontal diseases, but poorly for dental trauma. Forty percent of renal patients with renal colic did not report any pain relief after the treatment.

**Conclusion:**

Two minutes of pressure on both sciatic nerves can produce immediate significant conduction analgesia, providing a convenient, safe and powerful way to overcome clinical pain brought on by dental diseases and renal diseases for short term purposes.

**Trial registration:**

ACTR 12606000439549

## Background

At any given time, people may experience the pain of disease. In most cases, pain cannot be rapidly relieved. Overcoming pain in a fast and convenient way, therefore, is a worthwhile goal that has yet to be achieved despite considerable research into the subject. Presently, the analgesics available to patients are not satisfactory. For patients with acute initial pain, the time necessary to acquire appropriate pharmaceuticals, as well as, for drugs to reach effective levels, can mean hours or days of sustained pain before relief. Furthermore, many commonly used drugs, both over-the-counter and prescription, have well known serious side effects. For example, commonly prescribed NSAIDs can cause ulcers, gastrointestinal bleeding and renal impairment. Opioids can cause, among other things, constipation, nausea, vomiting, sedation, dependency, and addiction [1-4].

Many non-drug analgesics have been used to help manage pain: acupuncture, cryoanalgesia, transcutaneous electrical nerve stimulation (TENS), interferential stimulation (IFS), exercise, massage, music therapy, etc [5-7]. Current non-drug analgesics may not provide complete pain relief, or are applicable in limited circumstances, or only at pain centers. Their use alone or in combination with appropriate analgesic medications is an integral part of pain management in some pain centers. Literature reviews have documented the efficacy of some of these analgesics [5,8,9], but reveal conflicting results for others [10,11]. For example, TENS is used in a variety of clinical settings to treat different painful conditions [12-15]. However, the clinical effectiveness of TENS is controversial, with some studies supporting and others refuting its clinical use [11,16].

While applying traditional Chinese medicine by finger pressure stimulation of 'Chengfu' points located on the upper-back of the legs, we were surprised to find rapid relief of pain by the pressure stimulation. Because the 'Chengfu' point is anatomically associated with the sciatic nerve, further pilot studies were undertaken applying pressure along the sciatic nerves, but separate from the 'Chengfu' point, and, these produced the same results. Then, to evaluate the exciting finding, we designed the study reported here.

## Methods

### Setting

The clinical tests on renal patients were conducted between October 17, 2005 and January 24, 2006 in Anhui Province Hospital, Anhui Medical University, China. The clinical tests on dental patients were conducted between October 28, 2005 and January 24, 2006 in Tongling Hospital, Tongling, China, and between November 23, 2005 and April 12, 2006 in Chuzou Zhongxiyi Hospital, Chuzou, China. The study was separately approved by the ethic committees of each participating hospital – Anhui Province Hospital, Hefei, 230001, China (Approval data-June 2, 2005); Chuzou Zhongxiyi Hospital, Chuzou, 239000, China (approval data-May 28, 2005) and Tongling People Hospital, Tongling, 244000, China (approval data-April 11, 2005).

### Study design and procedure

This was a randomized, single-blind and placebo-controlled clinical trial on 66 participants out of 111 solicited patients. All the instructions and explanations were extended to patients of the "sciatic press" and "placebo press" groups equally. All the patients were told that the experiments were designed to test whether the method works for pain relief or not. All were advised that they could discontinue the experiment at any time without penalty, and their healthcare treatment would not be affected. After informed consent was obtained, the doctor or his assistant taught the patients how to evaluate pain using a visual analogue scale (VAS), with pain scaled from "0" for no pain to "10" for most pain. Thereafter, randomization of patients to the "sciatic press" group or the "placebo press" group was performed using Random Permuted Blocks. The three steps of the test were described to each patient, including: the baseline pain rating step, the leg pressure step for 2 minutes while lying down and, the post-pressure pain rating step for 10 min. In this context, the '0 minute' point indicated that the pain was estimated within one minute of discontinuing the leg pressure.

**Figure 1:**
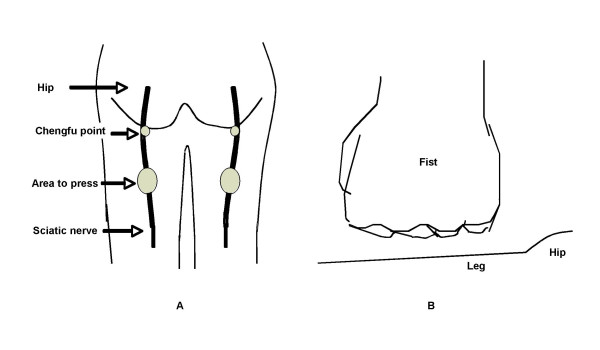
Chengfu point, Sciatic nerve pressure area and fist gesture.

The location of the classic 'Chengfu' point, the sciatic nerve pressure area, and the fist gesture for pressure application used are shown in Figure 1. For the "sciatic nerve press", 11 to 20 kg of pressure with each fist was applied to the sciatic nerve on the back of the thighs, while patients lay prone. For the "placebo press", the same amount of pressure was applied to a parallel spot on the front of the thighs, while patients lay supine. Doctors, using the gesture shown in the manuscript, pressed repeatedly on a balance to experience and learn how much force to use with each fist. The trained doctors determined how much force to apply based on the patients' body type. The heavily muscled and large body patients receiving greater pressure, (18–20 kg with each fist). Similarly, thin patients received less pressure (11–13 kg each fist). The ranges that were effective were determined in pilot studies.

Two minutes of pressure with the dorsal, proximal phalangeal surface of the fists was applied simultaneously to the sciatic, or the placebo location on both legs. Pain was then rated by patients, and, the value was recorded on a visual analogue scale table.

### Participants

All patients in pain from their respective pathologies during a clinic visit to Dental and Renal Clinics were eligible for the trial. Any patients who were younger than 15 years old, emotionally unstable, or receiving any analgesic within 12 hours of the test were ineligible for the study. All patients in the study had not been previously exposed to the method. Written informed consent was obtained from each participating patient.

Table I displays the patient groups and characteristics at inclusion in the study. Of the 111 solicited patients, 31 refused to join, 14 were considered ineligible, and, 66 patients ranging in age from 16 to 74 years participated in the study.

**Table 1:** Patients groups and Characteristics at Inclusion

	**Placebo Press **(n = 33)	**Sciatic Press **(n = 33)	p value
Test in Dental Diseases			
Participants (n)	21	21	-
Male (%)	71.4%	61.9%	0.513
Age	37.2(11.3)	36.6(13.4)	0.897
Baseline VAS	6.19 (1.47)	6.67 (1.59)	0.320
			
Test in Renal Diseases			
Participants (n)	12	12	-
Male (%)	58.3%	58.3%	1.0
Age	40.5(13.1)	52.4(16.9)	0.067
Baseline VAS	7.75 (2.01)	7.67 (1.97)	0.919

Age, gender and baseline VAS: mean (± SD), t-test.

The diseases in enrolled dental patients included dental caries, periodontal diseases and dental trauma. Renal diseases in recruits included kidney infections, stones and some other conditions.

### Statistical analysis

The baseline VAS scores and age of the participants were compared between the "sciatic press" groups and the "placebo press" groups by using t-tests. Categorical data were analyzed by using chi-square tests, or Fisher Exact tests. Changes, from the baseline for pain relief, were assessed by using paired *t*-tests, both for the "sciatic press" groups, and, the "placebo press" groups. Comparisons to the "placebo press" groups were performed using an analysis of covariance procedure, with adjustment for baseline VAS score, sex and age. All tests were two-sided, and, a p-value of < 0.05 was considered as statistically significant. All statistical analyses were performed with the use of SPSS statistical software (release 13.0).

## Results

**Figure 2:**
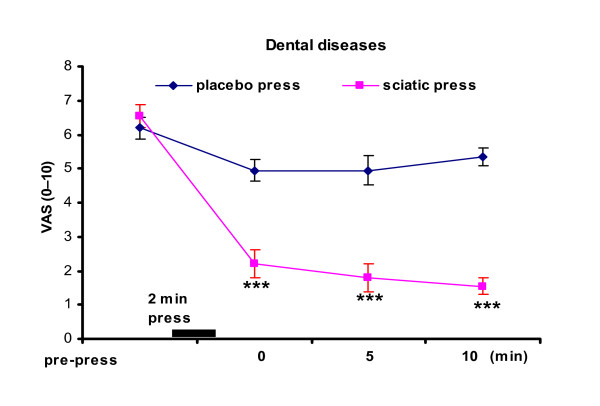
**Immediate relief of pain in dental patients**. *** p < 0.001, indicates significant difference between the "placebo press" and the "sciatic press". Results represent the mean (± SE).

Figure 2 displays the test in dental patients performed at two separate hospitals. Immediate pain relief by sciatic nerve press was 66.4%, versus relief of 20% with the placebo press. The significant relief of pain after the sciatic press was seen at all 3 time point of the 10 min period (p < 0.001 for all 3 time points). The results showed rapid relief of pain after the press for dental caries and periodontal diseases, but not for dental trauma.

**Figure 3:**
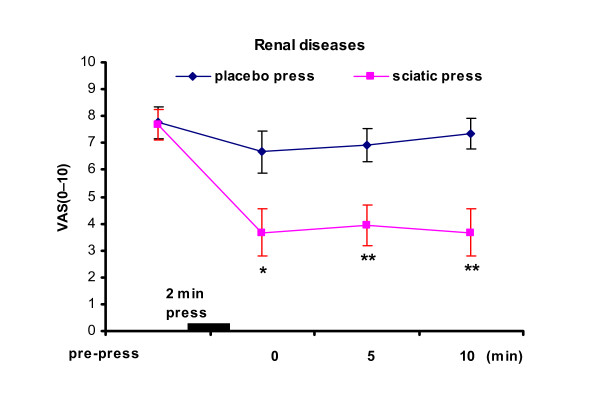
**Immediate relief of pain in renal patients**. * p < 0.05, **p < 0.01 indicate significant difference between the "placebo press" and the "sciatic press". Results represent the mean (± SE).

Next, we report that the sciatic press also produced rapid pain relief in patients with varying renal diseases. Figure 3 displays the results. The VAS scores at the 0, 5th, and 10th minute dropped significantly after sciatic nerve pressure. The immediate pain relief was 52.2% (p < 0.001) after the sciatic press, versus 14% relief after the placebo press. Notably, 40% of renal patients did not report any pain relief after the sciatic press in this test, and, these were all patients with the intense pain (three with VAS scores of 10, and two with scores of 8), and the pain of kidney colic.

## Discussion

The study was designed as a single blind rather than a double blind experiment; because, the method is highly effective in pain relief, and, doctors could easily identify the placebo or the actively treated during the test. The "Chengfu" point, located on the back of thigh just below the juncture of the hip (Figure 1), is recognized by practitioners of the discipline as related to urinary function (such as clarity of urine, frequency, etc) [17], but not to pain relief. There are five acupuncture points on the front, and five on the back of the thigh according to Chinese Traditional Medicine [17,18]. Usually only specialized doctors would know the specific functions for a given thigh point in China. To our knowledge, the pressure stimulation of any anterior or posterior thigh point for pain relief has not been reported. The exclusion criteria for this study specified that all patients in the study not have any previous exposure to the method. At entry to the randomization step, it was confirmed that all patients had no prior knowledge of, nor exposure to the method. Therefore, we believe that the blinding of patients was secure and unbroken under our tested protocol.

This method worked excellently on clinical pain from dental diseases, and part of renal diseases. The rapid pain relief in other diseases by the sciatic nerve press has been demonstrated in our further studies (manuscript soon to be submitted). No side effects were revealed in our study. Of those who declined to participate in the study, one of the most common reasons offered was skepticism of the method's simplicity. Some doctors participating in this study were also skeptical of the efficacy before the test.

Nearly 40% of renal patients did not report any pain relief after the "sciatic press"; these were all patients with the intense pain of kidney colic before the treatment. Similarly, the tests in dental patients showed excellent relief of pain for dental caries and periodontal diseases, but poor relief for dental trauma. However, limited by the small number of patients in this study, we can not assert whether the method works less effectively in intense pain states, or, if the loss of effect is related to the diseases' origin, or, to some other factor. To clarify this question, further systematic studies with a larger number of patients are needed.

Three critical factors determined the success of this method: the precise location, the total time, and, the suitable force for the applied pressure. Fists produced better results than finger tips in this method; because, is difficult to apply sufficient pressure for two minutes with finger tips, and, finger tips miss the precise sciatic nerve too easily. Pressure application was two minutes for all patients in this study. In our pilot study, for those patients who obtained pain relief with the method, 25% patients obtained relief within 30 to 60 second of the press, 54.7% patients between 60 to 90 seconds' after the press, and, the rest needed pressure for a longer time. Time shorter than 90 seconds of pressure for the press decreased the rate of success, and, the duration of pain relief declined proportionally. The third qualitative factor of importance is the amount of pressure applied. Insufficient force led to a failure of relief. The force used for the press was 11 to 20 kg from each fist.

This manipulation gave very distinct pain relief results in pilot studies and the clinical studies reported here. Based on the pilot studies, we did a prospective power analysis for dental diseases which gave a minimum sample size of 2 × 12 for the 10 min test. We didn't use the sample size as a strict limit for these two studies; because, pain relief with this method differed greatly between different diseases. The minimum sample size increased when the test period was lengthened. The result confirmed that the minimum sample size could be smaller for this method with some diseases for the 10 minutes observation period.

Stimulation of peripheral nerves elevated the pain threshold [19-22]. The phenomenon was suggested to be effective via multiple mechanisms [12,19,20,23,24]. One popular proposed mechanism is the Gate Control Theory of Pain [25], which proposes that stimulation of large-diameter afferent fibers can inhibit the transmission of nociceptive information, in the dorsal horn, to higher brain centers. The inhibition occurs rapidly, and is thought to involve the wide dynamic range (WDR) neurons [26-28]. The resulting analgesic effect is considered to be a short-lasting, segmental inhibition of pain [29-31].

The pinch press of rat sciatic nerve with a vascular clip (pinch force 120 g) caused attenuation of the WDR neurons' responses to various innocuous and noxious stimuli [32]. However, the study in cats with clip stimulation (pinch force 180 g) of the sciatic nerve noted an increased response of WDR neurons to the stimulation of the superficial peroneal nerve. Yet, the response of WDR was inhibited when using the low frequency stimulation (0.2 Hz) in the study [22]. The clip-pinch for the animal sciatic stimulation in the two reports, and the hand press for the human sciatic stimulation in this study, are both applied mechanical forces. However, the hand press stimulation is a much milder stimulus than the clips.

The Gate Control Theory of Pain can explain the rapid relief of pain by this method. However, the pain relief by this method is not limited to the segmental level only. Also, pain relief lasted only briefly for a small number of patients, while, more patients obtained longer relief periods, more than 30 minutes for a single two minute press (data not shown in this preliminary report). These observations suggest possible activation of multiple inhibitory systems. Another mechanism possibly involved is activation of the endogenous opioid system. In studying the effect of low frequency stimulation of rat sciatic nerve on long-lasting cardiovascular depression and pain threshold, Yao found that the pain threshold was increased by 50%. The analgesic effect he observed was antagonized by Naloxone suggesting the activation of the opioid system in the stimulation of the sciatic nerve [33].

The press stimulation, by itself, might cause pain, and such pain could activate a special form of descending pain inhibition called diffuse noxious inhibitory control (DNIC) [34-36], so called "pain inhibits pain". However, our method uses the smoother, dorsal proximal phalangeal surface of the fist to press on the thigh, not the pointed knuckles or finger tips. Only a small amount of discomfort was reported by a few patients in the study. Secondly, we used the same amount of pressure on the placebo patients as on the sciatic press patients, but it produced much less pain relief. Thirdly, more patients obtained longer relief periods with more than 30 minutes by the pressure (data not shown in this preliminary report), unlike the analgesia by DNIC which is known to be extremely short-lasting, ceasing within a few minutes.

This study reports a simple, immediately available and rapidly effective manipulation, hand stimulation of the sciatic nerve, to relieve pain of dental diseases and renal diseases. Anybody can apply it any time, any place, without a hospital setting.

## Conclusion

Two minutes of pressure on both sciatic nerves can produce rapid significant conduction analgesia, providing a convenient, safe and powerful way to overcome clinical pain brought on by dental diseases and renal diseases for short term purposes.

## Competing interests

The author(s) declare that they have no competing interests.

## Authors' contributions

JH had full access to all of the data in the study and takes responsibility for the integrity of the data and the decision to submit for publication. BW collaborated in the study design, participated in renal clinical tests and data analysis; WZ collaborated in clinical tests on dental patients and the data interpretation; GT collaborated in the study design, technical direction in the study and administrative support in dental clinical tests. All authors read and approved the final manuscript.

## Pre-publication history

The pre-publication history for this paper can be accessed here:


